# Small non-coding RNAs: key regulatory factors and potential therapeutic targets in tumor immunity

**DOI:** 10.3389/fimmu.2025.1639763

**Published:** 2025-09-11

**Authors:** Zihan Liu, Haotian Dong, Chengyuan Ye, Jianing Yan, Min Miao, Yongfu Shao

**Affiliations:** ^1^ Department of Gastroenterology, The First Affiliated Hospital of Ningbo University, Ningbo, China; ^2^ Health Science Center, Ningbo University, Ningbo, China

**Keywords:** small non-coding RNA, tumor immunity, immune checkpoint, tumor microenvironment, tumor immune escape, tumor immunotherapy

## Abstract

Tumor immunity has emerged as a focal point for cancer research. Although tumor immunotherapy represents a promising approach to cancer treatment, its effectiveness is often hindered by the heterogeneity of the tumor microenvironment (TME) and immune escape mechanisms. In recent years, small non-coding RNAs (sncRNAs) have attracted increasing attention in tumor immunology due to their essential role in gene regulation. This review systematically examines the multifaceted regulatory functions of sncRNAs in tumor immunity, with a focus on six major subtypes: microRNA, siRNA, piRNA, snoRNA, tsRNA, and snRNA. The molecular mechanisms by which these sncRNAs reshape the TME are discussed, including their roles in modulating immune cell differentiation (e.g., T cell polarization, macrophage phenotype transition), regulating immune checkpoint expression (PD-1/PD-L1, CTLA-4, Tim-3, LAG-3), and influencing tumor antigen presentation. This review also explores the dynamic network through which sncRNAs contribute to tumor immune escape. Furthermore, this study highlights the clinical potential of sncRNAs as liquid biopsy biomarkers and their application prospects in therapeutic strategies, such as targeted silencing of immunosuppressive molecules via nano-delivery systems, combination treatments with radiotherapy and chemotherapy, and Chimeric Antigen Receptor T-cell (CAR-T) therapy. Despite current challenges, including limited delivery efficiency and off-target effects, emerging technologies like AI-assisted sequence design and organ-on-a-chip models present new opportunities for clinical translation. This comprehensive review provides a theoretical foundation and translational insights for elucidating the functional network of sncRNAs in tumor immunology and advancing precise therapeutic interventions.

## 1 Introduction

The global incidence and mortality rates of tumors are increasing, posing a serious public health challenge that threatens human life and public health (1). Although conventional treatments such as surgery, radiotherapy, and chemotherapy have played essential roles in cancer management, they are often associated with significant limitations, including high recurrence rates and the development of strong drug resistance (2). In recent years, tumor immunotherapy has emerged as a promising alternative, offering renewed hope for cancer treatment (3). Tumor immunity involves a complex interplay between the immune system and tumor cells, encompassing processes such as immune cell recognition, tumor antigen presentation, and immune cell activation (4). The foundation of this process lies in maintaining a dynamic balance between the immune system and tumor cells. However, tumor cells can escape immune detection and destruction through various mechanisms (5), including impaired antigen presentation and the establishment of an immunosuppressive microenvironment, which collectively reduce the efficacy of immunotherapy (6).

Advancements in molecular biology research have highlighted the pivotal role of small non-coding RNAs (sncRNAs) in regulating gene expression within the context of tumor immunity. sncRNAs are short RNA molecules, typically ranging from 18 to 200 nucleotides (nt) in length, that do not encode proteins but exert important biological functions. Major subclasses include microRNA, siRNA, piRNA, snoRNA, tsRNA, and snRNA (7–10). Growing evidence indicates that sncRNAs play critical regulatory roles in tumor immunity, influencing the development, differentiation, and function of immune cells, as well as shaping the tumor immune microenvironment (TIME) (11–13). Studies have shown that sncRNAs can facilitate tumor immune escape and tumor progression by suppressing anti-tumor immune responses or promoting the accumulation of immunosuppressive cells within the TIME (14, 15). It has been demonstrated that sncRNAs are closely associated with the occurrence and progression of a variety of tumors. These findings enhance our understanding of the mechanisms underlying tumor immune escape and suggest new avenues for developing immunotherapeutic strategies centered on sncRNAs. Therefore, an in-depth investigation of the regulatory mechanisms and networks involving sncRNAs in tumor immunity is of great theoretical and clinical significance. Such efforts may uncover key molecular insights into immune escape and support the identification of novel targets for cancer immunotherapy (16). In this review, we have included all literature related to sncRNAs and tumor immunity, and elaborated on the regulatory roles of sncRNAs in tumor immunity as well as their potential therapeutic value.

## 2 Overview of small non-coding RNAs

**Figure 1:**
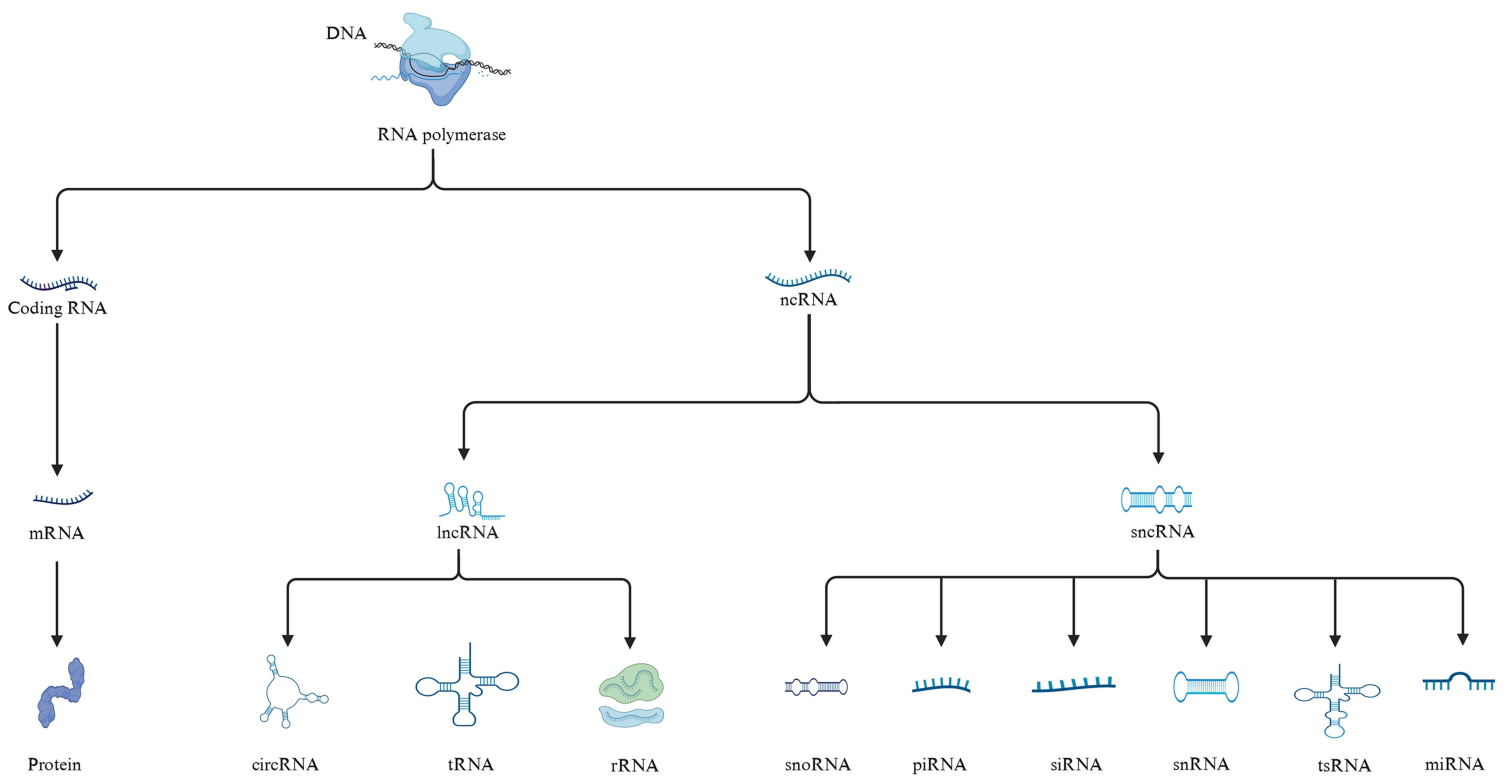
Classification of RNA. DNA is transcribed into coding RNA and non-coding RNA under the action of RNA polymerase. Coding RNA is further translated to generate proteins, while non-coding RNA is divided into lncRNA and sncRNA.

RNA plays a crucial role in biological processes. As shown in **Figure 1**, under the action of RNA polymerase, DNA is transcribed into coding RNAs and non-coding RNAs. Non-coding RNAs is further classified into long non-coding RNAs (lncRNAs, >200 nucleotides) and sncRNAs (≤200 nucleotides) according to size (9, 17). sncRNAs include microRNAs (miRNAs), small interfering RNAs (siRNAs), piwi-interacting RNAs (piRNAs), small nucleolar RNAs (snoRNAs), transfer RNA-derived small RNAs (tsRNAs), and small nuclear RNAs (snRNAs) (**Figure 1**).

microRNAs (miRNAs) are non-coding single-stranded RNA molecules approximately 20–24 nucleotides in length that primarily bind to the 3’ untranslated region (3′ UTR) of target mRNAs. When full complement is achieved, miRNAs mediate mRNA cleavage and degradation (18). In cases of partial complementarity, they inhibit translation, thereby regulating gene expression at the post-transcriptional level (19). miRNAs exert their functions not only within cells but also through microvesicles and exosomes (20). miRNAs are involved in the regulation of various biological processes, including cell proliferation, differentiation, and apoptosis (21, 22). Certain miRNAs exhibit tumor-promoting or tumor-suppressing activity, and both tumor cells and the TME exploit the aberrant expression of these miRNAs to drive self-remodeling through direct or indirect mechanisms (23). miRNAs are closely associated with tumor immunity, where they modulate immune cell function (24), participate in tumor immune escape, and influence the effectiveness of tumor immunotherapy (23).

siRNA are double-stranded RNA molecules consisting of 21–25 nucleotides. Based on their origin, siRNAs can be classified as endogenous, exogenous, or derived from post-transcriptional processing of short hairpin RNAs (shRNAs). Similar to miRNAs, siRNAs mainly act at the post-transcriptional level by guiding sequence-specific gene silencing (25, 26). They exhibit high sequence specificity and form RNA-induced silencing complexes (RISC) with specific enzymes and proteins within the cell (26). These RISC complexes recognize and bind to complementary mRNA sequences, leading to mRNA degradation or translation repression (27). Due to their negative charge and large molecular size, siRNAs are susceptible to degradation by serum endonucleases and displays poor cell membrane permeability. Therefore, efficient intracellular delivery typically requires a carrier system (28). Advances in nanotechnology have facilitated the development of novel delivery platforms capable of transporting siRNA into cells within the TME (29), thereby influencing the interactions between cancer and immune cells (30). For instance, Mitra Ghasemi-Chaleshtari and colleagues employed SPION nanoparticles loaded with siRNAs targeting PD-1 and A2aR, achieving substantial tumor growth inhibition and enhanced anti-tumor immune responses (27). Moreover, siRNA-based therapeutics have advanced into clinical trials. For instance, siRNA BMS-986263, a lipid nanoparticle-formulated siRNA designed to degrade HSP47 mRNA, has demonstrated promising results in clinical trials for treating advanced fibrosis (31). Several other clinical trials are currently underway, including NBF-006 for non-small cell lung cancer, pancreatic cancer (PC), and colorectal cancer (CRC), as well as EphA2 siRNA therapy for advanced or recurrent solid tumors (32).

piRNAs are typically 26–31 nucleotides in length and bind to the PIWI subfamily of Argonaute proteins to form the piRNA-Piwi complex (piRC) (33, 34). Their primary function is to maintain genomic stability in germ cells by suppressing transposon transcription and silencing transposable elements (33, 35). In addition to their role in genome defense, piRNAs indirectly influence tumor immune responses by regulating genes associated with tumor immunity (36, 37). piRNAs can bind to protein-coding mRNA sequences to modulate gene expression (38), and some are involved in antiviral defense mechanisms (39). Immunologically relevant piRNAs exhibit dynamic expression patterns during tumor progression and are associated with the prognosis and infiltration of various immune cells. For example, *in vitro* experiments have demonstrated that piR-021285 has been implicated in the development of breast cancer (40). piRNAs also serve as potential biomarkers for tumor diagnosis and prognosis, as well as for cancer subtype identification (35, 41). Researchers performed piRNA expression profiling in paired cancer and normal tissues and independently validated the findings in 771 CRC patients across three independent cohorts. They found piR-1245, which is overexpressed in CRC, is a novel oncogene. Its expression correlates strongly with tumor stage and metastasis, indicating its potential as a biomarker for CRC (42). Owing to their high target specificity, certain piRNAs can regulate immune checkpoint molecule expression and modulate anti-tumor immunity, offering promising avenues for precision cancer therapy (35, 43). piRNAs exert oncogenic or tumor-suppressive functions in cancer progression by regulating tumor cell proliferation, metastasis, drug resistance, and stemness, while their aberrant expression patterns with PIWI proteins in various malignancies offer novel biomarkers and therapeutic targets for tumor diagnosis and targeted therapy (44).

snoRNAs are small non-coding RNAs ranging from 65 to 300 nucleotides in length. Typically, they are processed from the intronic regions of small nucleolar RNA host genes (SNHGs) (45). Classic snoRNAs are categorized into three major groups: C/D box snoRNAs, H/ACA box snoRNAs, and small Cajal body-specific RNAs (scaRNAs) (33, 46). C/D box snoRNAs guide the 2′-O-methylation of ribosomal RNA (rRNA), whereas H/ACA box snoRNAs participate in pseudouridylation. scaRNAs are mainly involved in the modification of small nuclear RNAs (snRNAs) (33, 47). The principal function of snoRNAs is to regulate rRNA maturation. However, they are also involved in tumorigenesis and tumor progression (33, 46). snoRNAs can act as oncogenes or tumor suppressors and are implicated in the development of resistance to cancer therapies. For instance, SNORD11B enhances colorectal cancer progression by promoting tumor cell proliferation and invasion, inhibiting apoptosis via regulation of 2′-O-methylation. SNORD11B exerts a fine regulatory effect on T cell differentiation at the post transcriptional level by mediating the 2 ‘- O-methylation of let-7a. Its specific mechanism includes: this methylation modification enhances the stability of let-7a, prolongs its half-life by resisting degradation by nucleases to increase intracellular abundance. Simultaneously regulating the binding affinity between let-7a and target mRNAs such as key genes for T cell differentiation, thereby affecting the inhibitory effect on target gene expression. In addition, targeted regulation of cell cycle and differentiation related genes (such as IL-6, STAT3, etc.) by let-7a can indirectly affect related signaling pathways and regulate T cell fate determination (such as Th1/Th2 balance or Treg cell differentiation). Furthermore, methylated let-7a may compete with other non-methylated miRNAs for binding to RISC complexes, altering the global miRNA regulatory network and further affecting T cell differentiation. The specific target genes and pathways involved in the above mechanism still need further experimental verification (48). Moreover, snoRNAs have shown potential as biomarkers and therapeutic targets. For example, Researchers have conducted *in vivo* and *in vitro* experiments to demonstrate that small nuclear RNA host gene (SNHG5) may be a new target for treating gastric cancer. SNHG5, the host gene of snoRNA U50, inhibits gastric cancer progression by sequestering metastasis-associated protein 2 (MTA2) in the cytoplasm, indicating its therapeutic value in gastric cancer (49). snoRNAs also influence the TME by regulating the secretion of cytokines and chemokines, which affects immune cell recruitment and function. For example, SNORA38B promotes IL-10 secretion in tumor cells, leading to the recruitment of CD4+FOXP3+regulatory T cells and reduced infiltration of CD3+CD8+T cells in the TME of non-small cell lung cancer (NSCLC), thereby promoting tumor progression. Targeting SNORA38B using locked nucleic acids (LNAs) effectively suppresses NSCLC development and enhances sensitivity to immune checkpoint blockade therapy, making SNORA38B a potential therapeutic target for NSCLC (50).

tsRNAs are a class of non-coding small RNAs generated from the cleavage of tRNA molecules. They are generally categorized into two subtypes based on their cleavage sites: tRNA-derived stress-induced RNAs (tiRNAs) and tRNA-derived fragments (tRFs) (51–53). tiRNAs (28–36 nucleotides) are stress-induced fragments produced by specific cleavage at the anticodon loop of mature tRNAs, while tRFs (14–30 nucleotides) represent shorter fragments derived from either mature or primary tRNAs that can be generated under both normal cellular conditions and stress states, independent of specific physiological stimuli (51). tsRNAs participate in various biological processes, primarily through RNA interference and regulation of transcription and translation (53, 54). tsRNAs are closely linked to tumor immunity and are present in various bodily fluids—including blood, serum, urine, cerebrospinal fluid, and saliva—either freely or encapsulated in extracellular vesicles (10). This distribution allows tsRNAs to significantly influence intercellular communication within the TME. Aberrant expression of tsRNAs has been observed in numerous cancers (52, 53, 55–58). Their dysregulation is associated with tumor staging, lymph node metastasis, and poor clinical prognosis, indicating their potential as novel biomarkers for liquid biopsy (52, 53, 59, 60). For instance, a recent *in vitro* study has shown that tsRNA-49-73-GLU- CTC is highly expressed in the serum of patients with NSCLC. Inhibition of this tsRNA significantly reduces tumor cell proliferation and migration (61). Moreover, tsRNAs have emerged as promising targets for tumor therapy and drug-resistance treatment. Notably, tsRNA-Thr-5–0015 is markedly upregulated in the serum of patients with hepatocellular carcinoma (HCC), and its expression is closely correlated with TNM staging and lymph node metastasis. This tsRNA exhibits high stability and diagnostic precision, and its levels significantly decrease following surgical resection, establishing it as a potential biomarker for both hepatocellular carcinoma diagnosis and treatment (62). In CRC, tsRNA-GlyGCC is significantly overexpressed and is linked to enhanced cancer cell proliferation and resistance to 5-fluorouracil (5-FU) (54, 63). *In vitro* experiments have found that the tsRNA-GlyGCC promotes colorectal cancer progression and 5-FU resistance by targeting SPIB to regulate the JAK1/STAT6 signaling pathway (54). Inhibiting tsRNA-GlyGCC has been shown to increase the sensitivity of cancer cells to 5-FU, suggesting its utility as a combinatorial therapeutic target to improve treatment efficacy (54, 63).

snRNAs are typically 100–300 nucleotides in length and include several key types, such as U1, U2, U4, U5, and U6. These snRNAs associate with specific proteins to form small nuclear ribonucleoproteins (snRNPs), which together constitute the core components of spliceosomes (64). The primary function of snRNAs is to regulate the precise splicing of precursor mRNAs through spliceosome assembly, ensuring the accurate removal of introns and contributing to the diversity of gene expression (65). For example, U1snRNA identifies the 5’ GU sequence at intron sites and initiates splicing reactions via interaction with branch point sequences (66). Abnormalities in snRNA are closely linked to tumor immunity. For instance, mutations in U2 snRNA-related components, such as the SF3B1 gene, can lead to splicing errors that promote the progression of myelodysplastic syndromes to acute leukemia (67). Furthermore, certain snRNAs, such as U6, can function as innate immune sensors by activating the RIG-I-like receptor (RLR) signaling pathway, thereby inducing type I interferon secretion, enhancing CD8+T cell responses, and promoting anti-tumor immunity (68). Several studies have reported that U6 snRNA is upregulated in the serum and breast tissue of breast cancer patients (69), highlighting the association between snRNA dysregulation and tumor initiation and progression.

**Figure 2:**
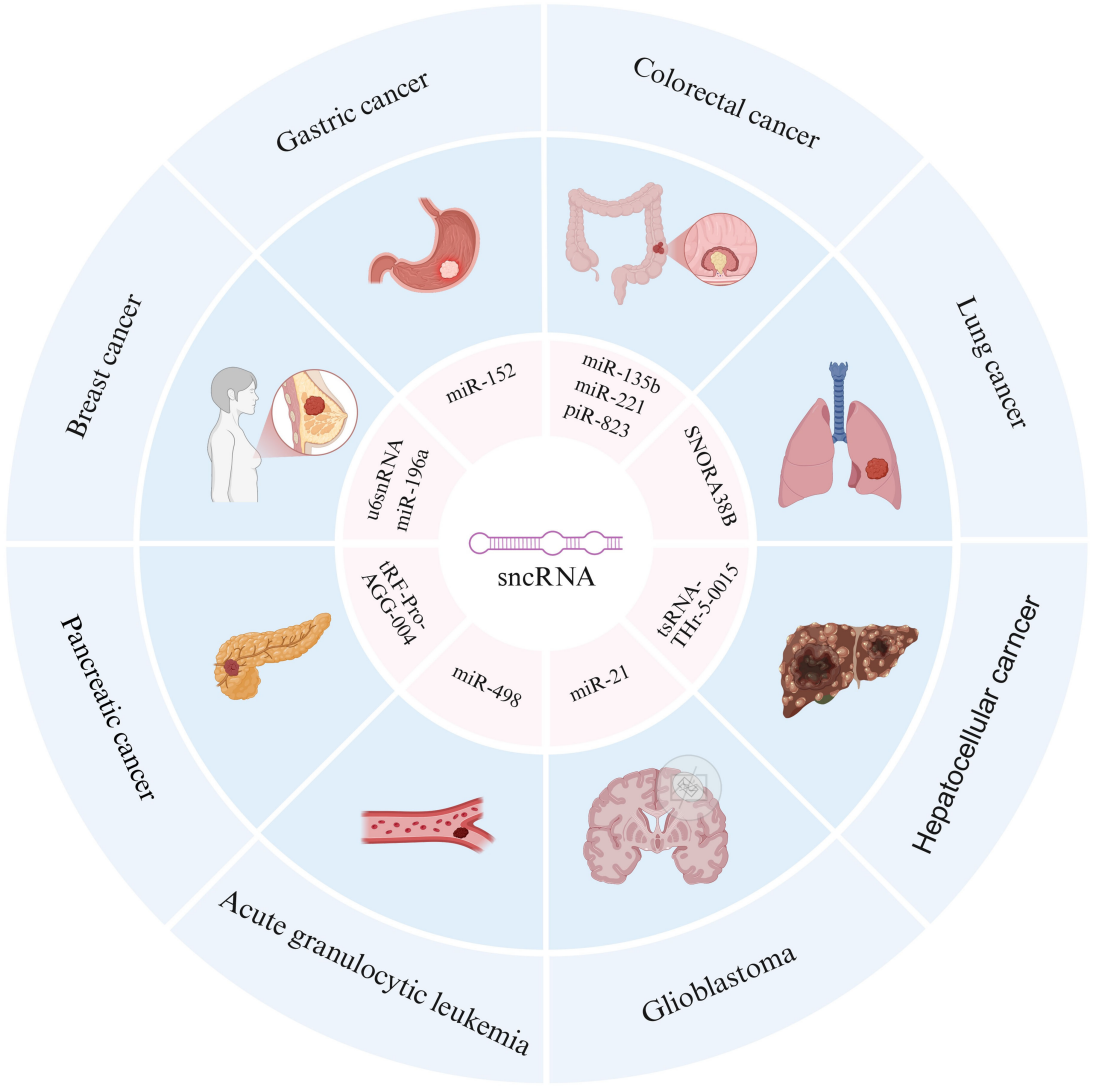
Association between sncRNAs and multiple cancers. The outer ring includes different types of cancers, including gastric cancer, colorectal cancer, lung cancer, hepatocellular cancer, glioblastoma, acute granulocytic leukemia, pancreatic cancer, and breast cancer. The inner loop annotates specific sncRNA molecules associated with these cancers, revealing the potential role of sncRNAs in the occurrence and development of different cancers.

In summary, it is evident that different types of sncRNAs exhibit significant differences in terms of size, structural characteristics, functions and mechanisms, as shown in **Table 1**. However, recent studies have found that their abnormal expression is closely related to human tumors such as gastric cancer, colorectal cancer, lung cancer, liver cancer, pancreatic cancer, breast cancer, acute leukemia, and glioma, revealing the potential role of sncRNAs in the occurrence and development of different cancers (**Figure 2**).

**Table 1:** The size, structural characteristics, functions and mechanisms of different types of small non-coding RNAs.

Category	Size	Structural characteristics	Functions	Mechanisms	References
miRNA	20–24 nt	Single-stranded non-coding RNA, processed from hairpin-structured precursors.	Regulate gene expression, incomplete complementary binding to the 3’ UTR of target mRNA, recruiting RISC complexes to inhibit translation or trigger mRNA degradation.	Forms the RISC with AGO proteins to recognize and bind target mRNAs.	(18, 19, 23)
siRNA	21–25 nt	Double-stranded RNA with relatively uniform length.	Mediates RNA interference and silences gene expression through degradation of target mRNAs.	Via complete complementary pairing, it recognizes and cleaves target mRNA, leading to its degradation.	(25–27)
piRNA	26–31 nt	Single-stranded RNA that associates with the PIWI protein family.	Predominantly silence transposons in germ cells and maintain genomic stability.	Bind with PIWI protein to form piRC, silencing the target sequence through DNA methylation or heterochromatin modification.	(33–38)
snoRNA	65–300 nt	Predominantly localized in the nucleolus and containing conserved sequence motifs.	Participate in methylation or pseudouridylation modification of rRNA.	Identifying target RNA through base pairing and guiding the action of modifying enzymes.	(33, 46–48)
tsRNA	18–40 nt	Cleavage fragments derived from tRNA.	Regulate translation, stress response, gene silencing.	Combining mRNA, ribosomes, or proteins to inhibit translation.	(51–60)
snRNA	100–300 nt	Rich in uridine, associating with specific proteins to form snRNPs.	Participate in pre mRNA splicing.	Binding with proteins to form snRNPs, participating in spliceosome assembly and splicing reaction.	(64–67)

## 3 Regulation of tumor immune cells and tumor immune microenvironment by sncRNA

### 3.1 Regulation of immune cell development, differentiation, and function

**Figure 3:**
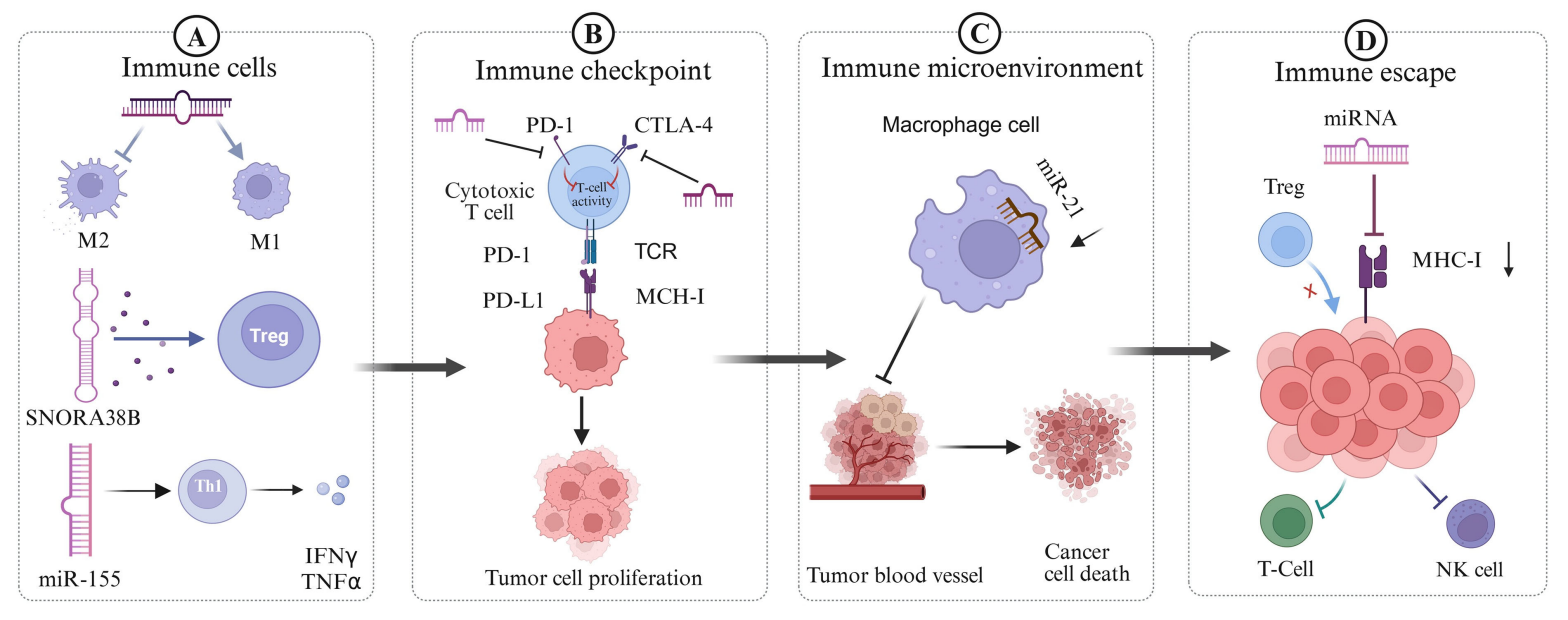
Four main ways for sncRNA to regulate tumor immunity. **(A)** Part A shows the effects of sncRNAs (such as miR-155, SNORA38B) on immune cells, which can promote M1 macrophage activation, inhibit M2 macrophage activation, and induce Th1 cells to secret cytokines such as INFγ and TNFα, promoting Treg cell. **(B)** Part B focuses on the regulation of tumor immune checkpoints by sncRNAs, such as certain miRNAs that can affect T cell activity by regulating PD-1 and CTLA-4 on the surface of cytotoxic T cells, thereby promoting tumor cell proliferation. **(C)** Part C indicates that sncRNA regulates the tumor immune microenvironment, such as the reduction and targeted inhibition of miR-21 expression in TAMs, which can lead to a decrease in tumor angiogenesis and promote cancer cell apoptosis. **(D)** Part D explains a mechanism of tumor immune escape, in which miRNA can regulate the expression of MHC-I on the surface of tumor cells and reduce their antigen presentation ability, thereby inhibiting Treg recognition of tumor cells and suppressing the activity of T cells and NK cells.

sncRNAs exert critical regulatory roles in the development, differentiation, and functional modulation of diverse immune cell populations involved in tumor immunity, including B cells, T cells, tumor-associated macrophages (TAMs), and natural killer (NK) cells (70–74). Several miRNAs influence T cell differentiation, particularly by promoting Th1 cell differentiation, thereby enhancing anti-tumor immune responses. For instance, miR-155 is expressed in multiple immune cell types and plays a pivotal role in polarizing primitive CD4+T cells into Treg, Th17, Th1, and Th2 cells (70, 72, 73). While miR-155 does participate in polarization, it does not “convert” CD4+ T cells equally toward all these subpopulations. Its role is better established in promoting Th1 and Th17 subsets, whereas its influence on Treg and Th2 is more ambiguous (74). Additionally, miR-155 regulates CD8+T cell activity and contributes to the differentiation and functional maturation of B cells (70). miR-155 also influences macrophage polarization by promoting the M1 phenotype while inhibiting the polarization and activation of M2 macrophages (73). This shift alters the inflammatory state of the TIME and reduces immune suppression (**Figure 3A**). Furthermore, miRNAs modulate NK cell development, proliferation, and activation by targeting key receptors and transcriptional regulators (75). Exosomal miRNAs are also involved in immune regulation. For example, in nasopharyngeal carcinoma, exosomal miR-24-3p suppresses T cell proliferation and differentiation by targeting fibroblast growth factor 11 (FGF11), thereby facilitating tumor immune escape and contributing to tumor pathogenesis (20). Relevant *in vivo* and *in vitro* experimental studies have suggested that snoRNAs may play an emerging role in regulating macrophage function through vesicle-mediated intercellular communication and epigenetic modifications (45, 76, 77). They may also influence macrophage polarization and cytokine production, thereby contributing to tumor development (45, 78).

### 3.2 The impact on the tumor microenvironment

The TME is a complex ecosystem composed of tumor cells, immune cells, stromal cells, lymphatic and blood vessels, and the extracellular matrix (79). Its immunosuppressive or immunostimulatory status directly affects tumor progression and therapeutic response (4, 79, 80). sncRNAs have emerged as key regulatory molecules within the TME by modulating the interactions between tumor and immune cells (81). Relevant animal experiments have shown that miR-21 in tumor-associated macrophages (TAMs) plays a regulatory role in immune responses. Targeted inhibition or downregulation of miR-21 in TAMs reduces tumor angiogenesis, enhances anti-tumor immunity, and promotes tumor cell death (**Figure 3C**) (82). Furthermore, sncRNAs contained in tumor-derived exosomes can be internalized by immune cells, altering their functional states and facilitating immune escape. For instance, in acute myeloid leukemia (AML), tumor cell-derived exosomes carrying miR-19a-3p are taken up by CD8+ T cells, leading to the suppression of effector molecule expression, T cell exhaustion, and subsequent immune escape (83). In addition, sncRNAs regulate the secretion of cytokines and chemokines by immune cells, thereby influencing their recruitment and infiltration and shaping the immunosuppressive or immunostimulatory state of the TME. miR-155 is essential for the expression of the IL-17F gene in Th17 effector cells. Its inhibition results in functional impairments of these cells. Given the role of Th17 cells and their cytokines in tumor immune regulation, such dysfunction may alter the inflammatory state and increase immunosuppression within the TME (84). Furthermore, as previously discussed, SNORA38B promotes the secretion of IL-10 by tumor cells, which recruit CD4+FOXP3+ regulatory T cells and reduce CD3+CD8+T cell infiltration in the TME of non-small-cell lung cancer. This activity facilitates the establishment of an immunosuppressive microenvironment, promoting tumor progression and weakening anti-tumor immunity (50).

## 4 Regulation of tumor immune checkpoints by sncRNAs and their involvement in tumor immune escape

### 4.1 Regulation of tumor immune checkpoint by sncRNAs

**Figure 4:**
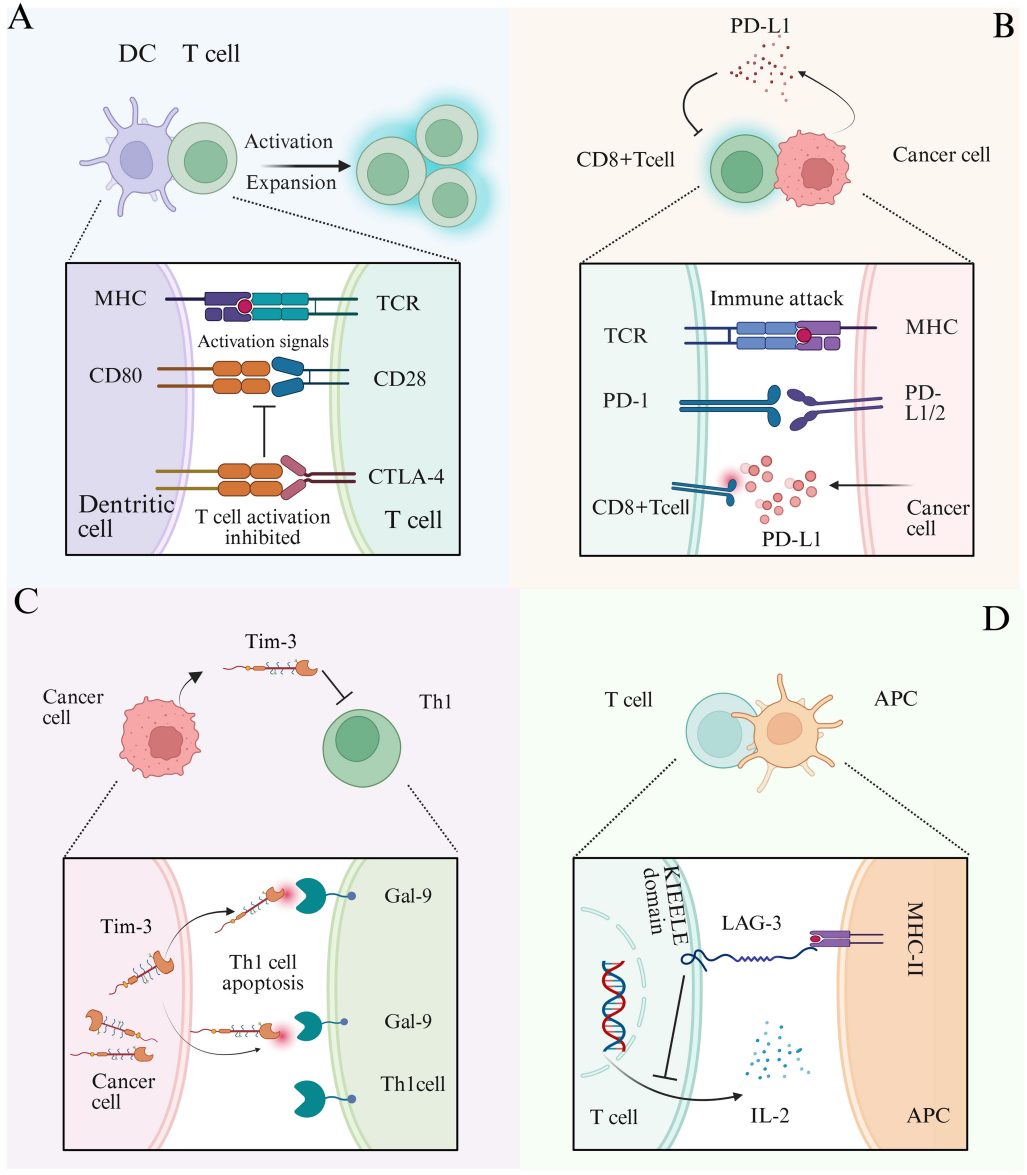
Schematic diagram of tumor immune checkpoint mechanism. **(A)** DCs interact with T cells, and DCs transmit activation signals to T cells through MHC and CD80 co-stimulatory molecules. When CTLA-4 binds to CD80 receptors, it competitively inhibits the binding of CD28 and CD80, thereby suppressing T cell activation. **(B)** CD8+T cells interact with cancer cells, and the T cell receptor (TCR) recognizes antigens presented by the MHC of cancer cells to initiate immune attacks. PD-1 on the surface of T cells binds to the receptor PD-L1/L2 on the surface of cancer cells, inhibiting immune attacks. PD-L1 secreted by tumor cells into the tumor microenvironment can reduce the activation of CD8+T cells and suppress immune responses. **(C)** When Tim-3 expressed by cancer cells binds to the ligand Gal-9 on the surface of Th1 cells, it can induce Th1 cell apoptosis and weaken their killing ability. **(D)** Interaction between T cells and antigen-presenting cells (APCs) involves the binding of the transmembrane protein LAG-3 (lymphocyte-activation gene 3) on T cells to MHC class II molecules on APCs. This interaction transmits an inhibitory signal via the intracellular KIEELE domain of LAG-3, suppressing the secretion of IL-2 and the function of T cells.

sncRNAs can enhance anti-tumor immunity by modulating the expression of immune checkpoint molecules. Immune checkpoints include a group of inhibitory receptors and ligands such as programmed death receptor 1 (PD-1) and its ligand PD-L1 (**Figure 4B**), cytotoxic T lymphocyte-associated antigen 4 (CTLA-4), T cell immunoglobulin and mucin domain-containing protein 3 (TIM-3), lymphocyte activation gene 3 (LAG-3), and T cell immunoglobulin and ITIM domain (TIGIT) (3, 85). When CTLA-4 binds to the receptors CD80 and CD86, it competes with CD28 for these ligands, thereby suppressing T cell activation (**Figure 4A**) (86). Immune checkpoint molecules serve as key facilitators of tumor immune escape, allowing tumor cells to evade immune detection and destruction. sncRNAs regulate the expression of checkpoint proteins, such as PD-1, PD-L1, and CTLA-4, thereby influencing T cell activity and immune response (**Figure 3B**). Ji Eun Won et al. developed a siRNA-based immune checkpoint silencing platform, the PLGA (PD-L1 siRNA+PD-1 siRNA) nanoparticle (NP) system, which was designed to inhibit PD-L1 expression in tumor cells. This strategy effectively restores CD8+T cell function and enhances anti-tumor immunity by suppressing immune checkpoint signaling in the TME (87). Further studies have shown that exosomes loaded with PD-L1 and CTLA-4 siRNAs significantly inhibit the proliferation of CRC cells, enhance apoptosis, and reduce immune escape capabilities (88). In addition to siRNAs, miRNAs regulate immune checkpoint expression. miRNAs directly target CTLA-4, thereby reducing its expression. For instance, miR-487a-3p directly targets CTLA-4, leading to reduced expression, whereas other miRNAs, such as miR-24 and miR-210, indirectly modulate CTLA-4 transcription by downregulating FOXP3, a key transcription factor involved in CTLA-4 expression (89–91).

Tim-3 and its receptor, galectin-9 (Gal-9), have emerged as novel targets in recent studies on tumor immune checkpoints (92). Tim-3, an inhibitory co-stimulatory molecule, is predominantly expressed in various immune cells and leukemia stem cells (93). Upon binding to Gal-9, Tim-3 induces apoptosis in Th1 cells, suppresses Th1 cell function (94), and contributes to immune tolerance and evasion (**Figure 4C**) (94, 95). This interaction (Tim-3/Gal-9) also triggers time-dependent metabolic changes in AML, influencing glucose and lipid metabolism to promote tumor progression. Consequently, Tim-3 has been recognized as a potential therapeutic target (93). Notably, cell function experiments have demonstrated that miR-498 effectively inhibits TIM-3 expression in AML cell lines, this miRNA thereby suppresses tumor cell proliferation (96). Lymphocyte activation gene 3 (LAG-3) is a type I transmembrane protein primarily expressed on the surface of T cells. Upon binding to MHC class II (MHC II) molecules, LAG-3 transmits inhibitory signals through its intracellular KIEELE domain, which blocks interleukin-2 (IL-2) secretion and suppresses T cell proliferation. The survival and immunosuppressive functions of regulatory T cells (Tregs) rely heavily on exogenous IL-2, thus, reduced IL-2 secretion leads to Treg depletion (**Figure 4D**). Furthermore, the inhibition of IL-2 limits the expansion of activated CD4+ and CD8+ T cells, thereby diminishing the adaptive immune response (97). miR-146 targets LAG-3 mRNA and downregulates its expression, alleviating T cell inhibition and enhancing anti-tumor immunity (98).

### 4.2 Participation of sncRNAs in tumor immune escape

Beyond regulating immune checkpoints, sncRNAs contribute to tumor immune escape through multiple mechanisms, including the modulation of tumor antigen presentation, reshaping of the tumor microenvironment (4), and regulation of immune checkpoint molecule expression (**Figure 3D**). Immunoregulatory miRNAs, such as miR-9, can alter the expression of major histocompatibility complex (MHC) molecules on the surface of tumor cells, thereby reducing their antigen-presenting capacity and enabling evasion from T cell recognition (99). Tumor-derived miRNAs may be encapsulated in exosomes or microvesicles and transferred to tumor-infiltrating lymphocytes, promoting an immunosuppressive TME that facilitates immune escape (100). For instance, piR-has-30937, derived from the extracellular vesicles of pancreatic neuroendocrine tumors, enhances CD276 expression in macrophages via the PTEN/AKT signaling pathway. This upregulation suppresses T cell-mediated anti-tumor responses and promotes tumor immune escape (101). sncRNAs also contribute to the creation of an immunosuppressive microenvironment by influencing tumor cells or tumor-associated macrophages to secrete inhibitory cytokines, such as transforming growth factor-β (TGF-β) and interleukin-10 (IL-10). These factors impair immune cell function and support tumor immune escape (102).

**Figure 5:**
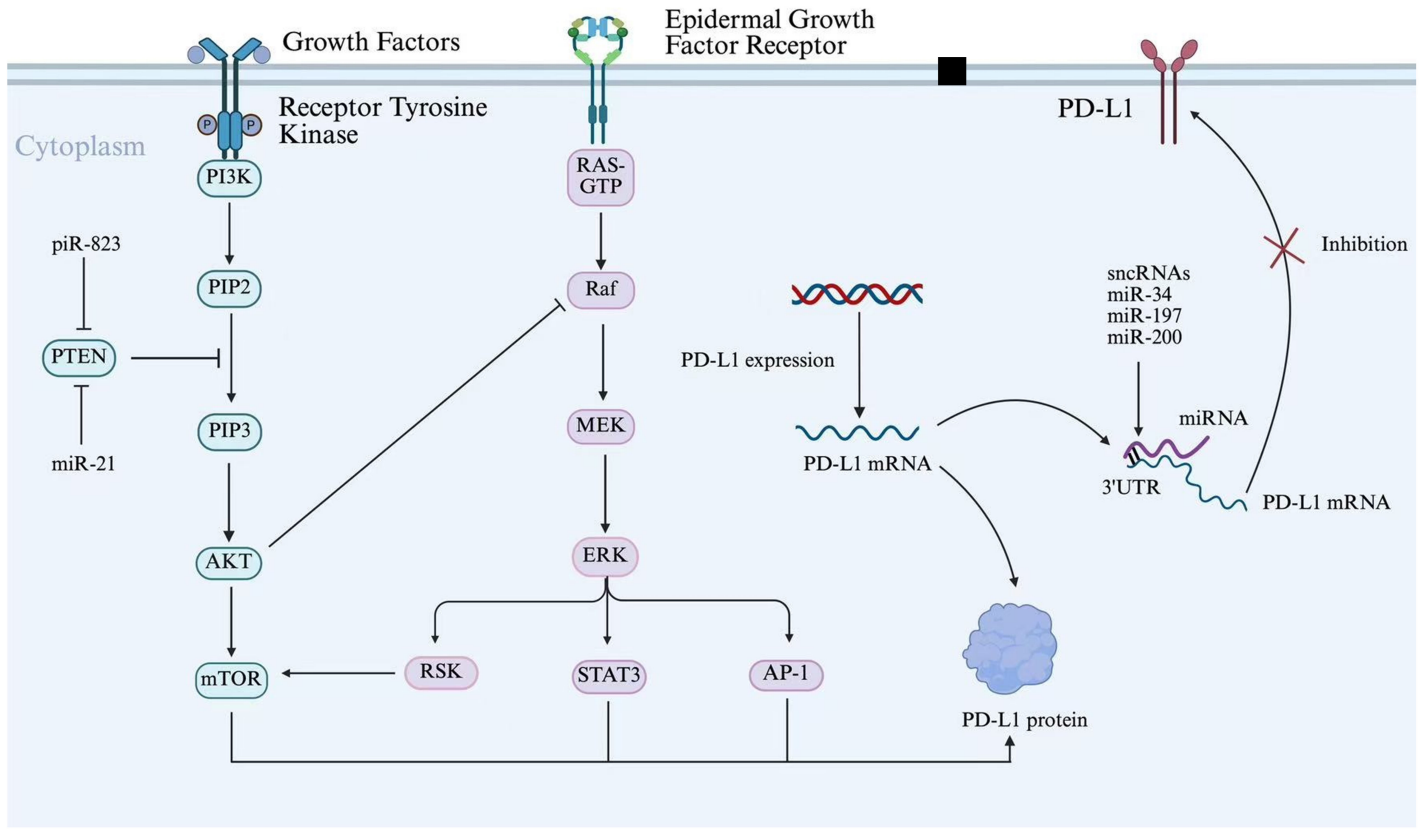
sncRNAs regulate PD-L1 expression through the shared PI3K/AKT signaling pathway. Schematic diagram illustrating the signaling pathways regulating PD-L1 expression and function. Growth factors activate Receptor Tyrosine Kinase, initiating the PI3K/AKT/mTOR and RAS/Raf/MEK/ERK cascades. These pathways converge on transcription factors (STAT3, AP-1) to drive PD-L1 transcription. Additionally, post-transcriptional regulation is depicted, with sncRNAs (miR-34, miR-197, miR-200) targeting PD-L1 mRNA 3’ UTR.

Moreover, sncRNAs not only act directly through epigenetic mechanisms but also exert regulation at the post-transcriptional level, which influences epigenetic expression (103). For instance, miR-142-3p, present in exosomes from M1 macrophages, targets high mobility group box 1 (HMGB1) and influences tumor immune escape in glioblastoma by modulating the PD-1/PD-L1 checkpoint (104). In CRC, miR-21 suppresses genes that negatively regulate PD-L1 expression, including PTEN, and activates the PI3K/AKT signaling pathway. Furthermore, miR-21 promotes PD-L1 expression by targeting and suppressing PDCD4 (programmed cell death protein 4), thereby relieving its inhibitory effect on the PI3K/AKT pathway (105). This leads to enhanced membrane recruitment and phosphorylation-mediated activation of PI3K. The activated PI3K catalyzes the conversion of PIP2 to PIP3, which recruits AKT (protein kinase B) to the plasma membrane through its PH domain, where it undergoes phosphorylation by PDK1 and mTORC2 for full activation. The activated AKT subsequently upregulates PD-L1 transcription and translation by phosphorylating downstream transcription factors (e.g., NF-κB, STAT3) or directly modulating mTORC1 signaling. This cascade leads to elevated levels of PD-L1, which, upon binding to PD-1 receptors on T cells, significantly reduce T cell activation and proliferation, thereby enabling tumor cells to escape immune surveillance (105–107). Notably, certain sncRNAs can regulate PD-L1 expression through shared signaling pathways. For instance, both miR-21 and piR-823 enhance PI3K/AKT/mTOR signaling activity by suppressing PTEN expression, thereby promoting PD-L1 upregulation and promoting tumor immune escape (108). Additionally, miR-34, miR-197, and miR-200 collectively target the 3’ UTR of PD-L1 mRNA to enforce post-transcriptional silencing, thereby suppressing PD-L1 protein synthesis (**Figure 5**) (109). This downregulation ultimately inhibits PD-L1-mediated immune escape. These intertwined regulatory networks illustrate the complexity of sncRNAs-mediated immune escape, suggesting that dissecting their crosstalk with PD-L1 and signaling pathways could unveil novel therapeutic vulnerabilities.

## 5 Application prospects of sncRNA in tumor immunotherapy

### 5.1 sncRNA as a tumor biomarker

**Figure 6:**
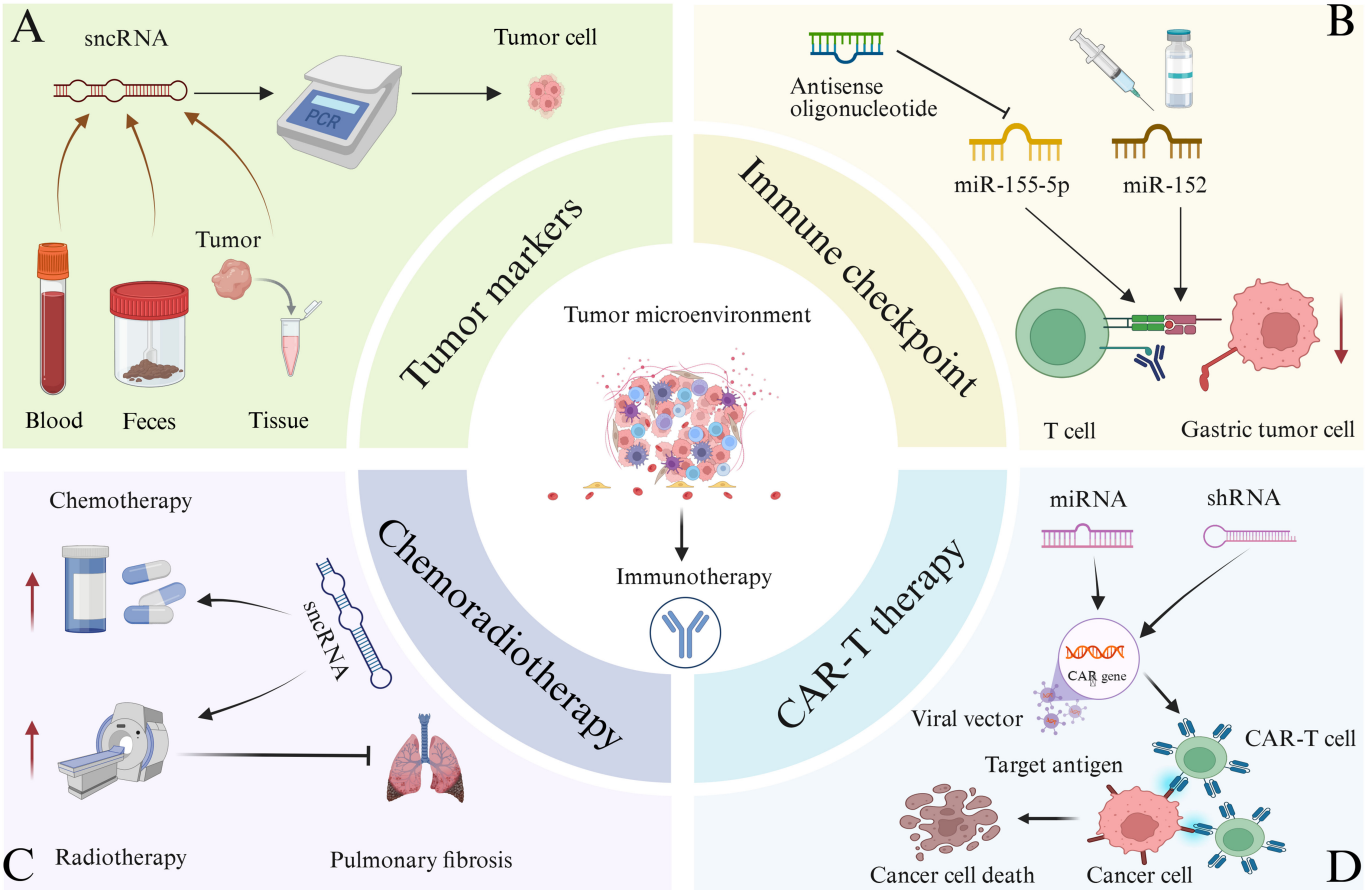
The relevant applications of sncRNA in tumor immunotherapy are illustrated. **(A)** Quantitative PCR and other techniques can be used to detect significant abnormal expression of certain sncRNAs in the blood, feces, and tumor tissues of certain cancer patients. Therefore, sncRNAs can function as tumor biomarkers and assist in tumor diagnosis. **(B)** Targeting immune checkpoints for treatment. The function of antisense oligonucleotides in inhibiting miR-155-5p, leading to a decrease in abnormal expression of immune checkpoint molecules, enhancing T cell function, and facilitating the clearance of gastric cancer cells. miR-152 can directly bind to the 3’ end translation region of B7-H1 in gastric cancer cells, thereby inhibiting its expression. Therefore, relevant drugs targeting sncRNA and tumor immune checkpoints can be developed for immunotherapy of tumors. **(C)** Combination of sncRNA with radiotherapy and chemotherapy for the treatment of tumors. In addition, certain sncRNAs combined with radiotherapy can alleviate lung injury and pulmonary fibrosis caused by radiotherapy alone. **(D)** Combination of sncRNA and CAR-T therapy for tumor treatment. CAR-T cells are generated by introducing CAR genes into T cells through viral vectors, targeting cancer cell antigens, and inducing cancer cell death. Introducing miRNA or shRNA into CAR-T cells may enhance the anti-tumor efficacy of CAR-T cell therapy.

sncRNAs have emerged as critical targets in tumor biomarker research, owing to their exceptional stability in body fluids and tissue samples, tissue-specific expression patterns, and strong association with various diseases (62, 110–113). These characteristics make sncRNAs highly suitable for non-invasive detection, facilitating early tumor screening. Moreover, their dynamic expression profiles are closely linked to disease progression and therapeutic response, offering substantial value in clinical diagnostics and prognostic evaluations (113, 114). Some sncRNAs are abnormally expressed in colorectal tumors and remain stable in blood and feces. Their expression levels can be quantified using methods such as quantitative PCR and high-throughput sequencing (**Figure 6A**) (114). For instance, elevated levels of miR-196a have been detected in drug-resistant estrogen receptor (ER)-positive breast cancer, while high miR-10b expression has been observed in ER-negative breast cancer, indicating their potential as subtype-specific biomarkers (113). Multiple studies have demonstrated that distinct miRNA expression profiles can be consistently detected in CRC tissues and fecal samples. Wu et al. conducted a microarray analysis of fecal miRNAs and found that miR-135b was significantly upregulated in CRC and advanced adenomas. Notably, its expression decreased significantly in postoperative fecal samples, highlighting its potential as a non-invasive biomarker for the clinical diagnosis and monitoring of CRC and advanced adenomas (115). Additionally, miR-221 has been identified as a potential biomarker for CRC diagnosis. Notably, miR-221 levels in feces exhibit higher specificity than those in plasma and are not influenced by antibiotic use (116). In addition to miRNAs, other sncRNA subtypes have also shown promise in CRC biomarker research. For example, in a clinical observational study analyzing serum and tissue samples from patients with CRC, piR-823 has been found to be upregulated in the serum and tissues of patients with CRC, and its expression levels are positively correlated with clinical staging. Higher expression levels are associated with more advanced stages, supporting that piR-823 could serve as a potential diagnostic biomarker for CRC, while its correlation with clinical staging preliminarily suggests potential prognostic value (117). Furthermore, tsRNAs have also demonstrated utility as biomarkers in various cancers. For instance, researchers demonstrated that serum tRF-Pro-AGG-004 and tRF-Leu-CAG-002 can serve as novel and promising biomarkers for PC diagnosis by integrating multi-omics technologies with clinical samples, cellular models and animal models. Subsequent randomized clinical trials are required to evaluate the potential application of the two-tsRNAs signature in the early diagnosis and prognosis of PC (118). In addition, the expression levels of tRNA-ValTAC-3, tRNA-GlyTCC-5, tRNA-ValAAC-5 and tRNA-GluCTC-5 are significantly increased in the plasma exosomes of patients with HCC, indicating that tsRNA in plasma exosomes may serve as potential biomarkers for tumor diagnosis (114).

Similarly, sncRNAs can also serve as biomarkers for non-solid tumors. Studies have comprehensively characterized the landscape of circulating sncRNAs in serum and bone marrow supernatants from patients with AML using high-throughput sequencing. The combination of tsRNA signatures (e.g., tsRNA^Ala-TGC^, tsRNA^Gly-CCC^) screened by machine learning models exhibited superior diagnostic efficacy (AUC > 0.95) compared with miRNAs in distinguishing AML patients from healthy individuals, and their expression levels were associated with AML risk stratification. Furthermore, sncRNAs profile derived from peripheral blood and bone marrow showed a high degree of concordance, suggesting that serum tsRNAs can serve as non-invasive biomarkers to replace bone marrow tests, thus providing a novel strategy for the early diagnosis and prognostic evaluation of AML (119).

### 5.2 Targeting immune checkpoint pathways

Immune checkpoints play a critical role in tumor immune escape, and their blockade has become a key strategy in tumor immunotherapy (84, 120). Several immune checkpoint inhibitors (ICs) have been approved for clinical use, including the PD-1/PD-L1 inhibitor Nivolumab and Pembrolizumab, the LAG-3 inhibitor Relatlimab and the CTLA-4 inhibitor ipilimumab, all of which are used in the treatment of advanced melanoma (121–124). Tiragolumab, an inhibitor targeting TIGIT, is currently undergoing clinical evaluation (125). Moreover, the combination of relatlimab and nivolumab has demonstrated synergistic anti-tumor activity (126). sncRNAs can modulate immune checkpoint expression through diverse mechanisms, offering novel therapeutic approaches for cancer treatment (85, 89). As previously noted, miR-155 is a key regulator of tumor immune response. Specifically, miR-155-5p can inhibit PD-L1 expression by binding to the 3’ UTR of its mRNA, thereby modulating CD8+T cell function (**Figure 6B**) (127). The development of therapeutic strategies targeting miR-155 represents a promising approach for tumor immunotherapy. One such strategy involves the use of antisense oligonucleotides to inhibit miR-155 activity, which may lead to a reduction in aberrant immune checkpoint expression, restoration of immune cell function—particularly that of T cells—and enhancement of the host’s anti-tumor immune response (128). A study integrating *in vitro*, *in vivo*, and clinical trial investigations demonstrated that the miR-155 oligonucleotide inhibitor Cobomarsen reduced cell proliferation and induced apoptosis in activated B-cell–like diffuse large B-cell lymphoma (ABC-DLBCL) cells, and exerted no toxic effects on the enrolled patient who underwent five cycles of Cobomarsen treatment (129). siRNA can selectively bind to complementary sequences of immune checkpoint mRNAs, thereby silencing the expression of corresponding immune checkpoint genes. *In vitro* studies have shown that nanocarrier systems significantly enhance the cellular delivery of siRNA, enabling the precise targeting and degradation of mRNAs encoding immune checkpoint molecules such as CTLA-4, PD-1, and PD-L1. This targeted silencing disrupts inhibitory signaling among tumor cells, T cells, macrophages, and dendritic cells, effectively reducing the immunosuppressive TME. As a result, T cell function is reactivated, leading to improved tumor recognition and clearance (130–132). In addition, studies from both *in vitro* experiments and *in vivo* trials have shown that 3p-GPC-3 siRNA enhances the activation of CD4+ T cells, CD8+T cells, and natural killer (NK) cells, while reducing the presence of regulatory T cells within the TME. When combined with PD-1 blockade, 3p-GPC-3 siRNA demonstrates a significantly enhanced anti-tumor effect and markedly inhibits the growth of HCC (131). The immune checkpoint molecule B7-H1 functions as a ligand for PD-1, and their interaction suppresses T cell function and immune responses. A strong correlation has been observed between miR-152 and B7-H1 mRNA levels in gastric cancer tissues. miR-152 directly targets the 3′ UTR of B7-H1 mRNA in gastric cancer cells to inhibit its expression, which promotes T cell function and enhances the anti-tumor immune response by blocking the B7-H1/PD-1 signaling pathway, ultimately reducing immune escape (**Figure 6B**). Notably, reduced expression of miR-152 is associated with increased tumor size and a higher rate of lymph node metastasis, suggesting that miR-152 may serve as a potential prognostic marker and therapeutic target in gastric cancer (133). Furthermore, sncRNAs and small-molecule inhibitors targeting other immune checkpoints, such as LAG-3 and TIM-3, are under active investigation in both preclinical studies and clinical trials, offering promising potential for expanded applications in tumor immunotherapy (125, 134).

### 5.3 Combination therapy

The integration of sncRNA with conventional treatment modalities, such as chemotherapy, radiotherapy, and CAR-T therapy, has significant therapeutic potential.

#### 5.3.1 SncRNA combined with chemotherapy

Combining sncRNA-based therapy with chemotherapy has demonstrated synergistic effects in enhancing anti-tumor immunity (**Figure 6C**) (135–138). For example, the use of siRNA targeting PD-L1 in conjunction with chemotherapy can effectively modulate the immunosuppressive TME, thereby amplifying the response to chemotherapy-induced immunogenic cell death. Furthermore, siRNA co-delivered with chemotherapeutic agents can inhibit genes involved in tumor growth and progression, resulting in suppressed tumor proliferation (136, 138–141). Researchers synthesized a combined silica nanoparticle system loaded with siRNA and doxorubicin (DOX), aiming to reverse the drug resistance of DOX-resistant KB-V1 cervical cancer cells. *In vitro* experiments, they specifically downregulated genes associated with the activation of P-glycoprotein (P-gp) pumps via siRNA, successfully reinducing drug-resistant cancer cells into a sensitive state. This strategy effectively increased the intracellular accumulation concentration of DOX, thereby enhancing the anticancer activity of the drug (139). Moreover, siRNA can be used to target genes associated with chemotherapy resistance, thereby overcoming or reducing drug resistance and enhancing the overall anti-tumor response (142). Researchers investigated the impact of silencing the Bcl-2 gene via small interfering RNA (siRNA, namely siBcl-2) on the efficacy of 5-FU in colorectal cancer. The results showed that the combined application of siBcl-2 liposomes and the 5-FU prodrug S-1 could inhibit tumor growth in the DLD-1 xenograft model, and a dual-modulation strategy for addressing 5-FU-resistant tumors was proposed. In this context, the co-delivery of siRNA and chemotherapeutic agents via nanocarrier systems represents a promising approach to improve therapeutic efficacy and combat drug-resistant cancer cells (143). In addition, miRNAs have emerged as therapeutic targets in osteosarcoma. The targeted delivery of miRNA using nonviral carriers—such as polymers, lipid nanoparticles, exosomes, and inorganic nanoparticles—has shown potential for osteosarcoma treatment. When combined with chemotherapeutic agents, this approach can enhance the sensitivity of osteosarcoma cells to chemotherapy and significantly improve treatment outcomes (144). Furthermore, studies have shown that combining miR-21 inhibitors with chemotherapeutic agents, such as paclitaxel, can effectively inhibit the proliferation of human glioblastoma cells (145).

#### 5.3.2 sncRNA combined with radiotherapy

Radiotherapy induces the release of tumor-associated antigens, and sncRNA can enhance the recognition and response of immune cells to these antigens, thereby synergistically amplifying the anti-tumor effects of radiotherapy (**Figure 6C**) (146). For instance, miR-101 targets two key DNA repair genes, dependent protein kinase catalytic subunit (DNA PKcs) and ataxia telangiectasia mutated (ATM), by binding to their 3′ UTRs. Upregulation of miR-101 reduces the expression of these proteins, impairing the DNA repair capacity of tumor cells and increasing their sensitivity to radiation-induced damage (147). Similarly, siRNAs can precisely silence genes associated with radioresistance, such as ATM. In preclinical studies using glioma cell lines and xenograft models, inhibiting such genes via siRNA-based strategies has been shown to effectively enhance tumor radiosensitivity, as demonstrated in gliomas (148). In addition to enhancing radiosensitivity, sncRNAs exert protective effects on normal tissues during radiotherapy. Certain sncRNAs have been shown to reduce local inflammatory responses and mitigate or reverse fibrosis by modulating inflammation-related signaling pathways. For example, miR-486-RBD-MSC-Exo, a construct based on miR-486-5p, can inhibit fibrosis in lung epithelial cells and alleviate radiation-induced lung injury and fibrosis (**Figure 6C**) (149, 150).

#### 5.3.3 sncRNA in combination with CAR-T therapy

sncRNA can optimize the function of CAR-T cells and enhance their cytotoxic activity against tumor cells (**Figure 6D**). While CAR-T therapy is primarily employed in the treatment of hematological malignancies, it has demonstrated promising potential in targeting solid tumors (151–155). Previous studies have shown that the co-transfection of CAR-encoding mRNA and siRNA can improve CAR-T cell efficacy by downregulating inhibitory receptors such as PD-1 and CTLA-4. *In vitro* experiments have demonstrated that this strategy may represent a novel approach to augment CAR-T cell-mediated immunotherapy (156). Furthermore, miR-153 has been shown to enhance the therapeutic efficacy of CAR-T cells by suppressing the expression of indoleamine 2, 3-dioxygenase 1 (IDO1) in colon cancer cells (157). More recently, a research team proposed the integration of miRNA or shRNA into retroviral vectors used for CAR expression. Using this approach, they developed anti-CD19 CAR-T cells with upregulated miR-155 expression, which exhibited robust anti-tumor activity both *in vitro* and *in vivo*. These findings suggest that the incorporation of miRNA or shRNA into CAR-T cell constructs may further enhance the therapeutic efficacy of CAR-T cell therapy (158).

At present, there are many clinical trials of sncRNAs in tumor immunotherapy. For example, researchers have attempted to apply CART cell therapy to other malignant tumors. In order to prevent allogeneic CART cell graft-versus-host disease (GvHD), researchers chose a shRNA based on miRNA, which targets CD3ζ and effectively downregulates the expression of T cell receptor below the detection level. They generated allogeneic anti-B cell mature antigen CART cells (CYAD-211), which co expressed shRNA based on anti-CD3ζ miRNA in CAR construction *in vivo*, effectively inhibited TCR mediated signaling *in vitro*, and effectively inhibited GvHD *in vivo*. Subsequently, in a phase I clinical trial (NCT04613557), CYAD-211 was evaluated in patients with relapsed or refractory multiple myeloma, confirming its good safety. The clinical trial results showed no signs of GvHD and indicated effective downregulation of TCR (159). In addition, Lna-i-mir-221, an inhibitor of oncogenic miRNA, showed outstanding performance and high safety in the phase I trial of refractory advanced solid tumors (NCT04811898), and 50% of patients achieved disease control. One patient with colorectal cancer achieved partial remission lasting over three years. In addition, pharmacodynamics confirmed that miR-221 down regulated and activated tumor suppressor targets CDKN1B/p27 and PTEN. This study is the first human trial of LNA miRNA inhibitor and has promoted the phase II clinical development of solid tumors (160). In summary, there are still many ongoing clinical trials on the application of sncRNAs in tumor immunotherapy, and sncRNAs based therapies have the potential to change tumor treatment.

## 6 Research challenges and future prospects

In summary, sncRNAs play an important regulatory role in tumor immunity. Different types of sncRNAs play different roles in the occurrence, development, and immune escape of various tumors. Certain microRNAs can participate in tumor immune escape by influencing tumor cell apoptosis, invasion, metastasis, and the function of immune cells in the TME. siRNAs are capable of targeting specific molecules to modulate immune cell activity and enhance the body′s anti-tumor immune response. Additionally, other sncRNAs including piRNAs, snoRNAs, and tsRNAs each play distinct roles in regulating tumor immunity-related pathways or cellular functions. Small non-coding RNA molecules and their roles in tumor immunity are summarized in **Table 2**.

**Table 2:** Small non-coding RNA molecules and their roles in tumor immunity.

Molecular type	Exemplar molecule	Tumor type	Key role in tumor immunity	References
microRNA	miR-196a	Breast cancer	Alters apoptosis and angiogenesis	(112)
microRNA	miR-10b	Breast cancer	Cell invasion and metastasis inductor	(112)
microRNA	miR-19a-3p	AML	T cell exhaustion and immune escape	(83)
microRNA	miR-135b	CRC	Potential noninvasive biomarker	(114)
microRNA	miR-221	CRC	Potential biomarker for colorectal tumors	(115)
microRNA	miR-152	Gastric cancer	Inhibiting the B7-H1/PD-1 pathway, and improve immune escape	(130)
siRNA	3p-GPC-3 siRNA	HCC	Enhance the activation of CD4+, CD8+ T cells and NK cells	(130)
siRNA	PLGA (PD-L1 siRNA+PD-1 siRNA) - NP	Cervical cancer Lymphoma	inhibit the expression of PD-L1 in tumor cells	(87)
piRNA	piR-021285	Breast cancer	Associated with the prognosis and infiltration of various immune cells	(40)
piRNA	piR-1245	CRC	Potential biomarker of CRC	(42)
piRNA	piR-hsa-30937	Pancreatic tumor	Enhance CD276 expression in macrophages via the PTEN/AKT pathway	(100)
piRNA	piR-823	CRC	Screening diagnostic biomarker for CRC	(116)
snoRNA	SNORD11B	CRC	Regulating 2′-O-methylation modification	(48)
snoRNA	snoRNA U50	Gastric cancer	Isolates metastasis associated protein 2	(49)
snoRNA	SNORA38B	NSCLC	Promote tumor progression	(50)
tsRNA	tsRNA-49-73-GLU-CTC	NSCLC	Reduce the proliferation and migration ability of tumor cells	(61)
tsRNA	tsRNA-Thr-50015	HCC	Establishing it as a biomarker for both the diagnosis and treatment of HCC	(62)
tsRNA	tsRNA-GlyGCC	CRC	As a new target in combination with 5-FU to form more effective treatment plans	(54, 63)
tsRNA	tRF-Pro-AGG-004	Pancreatic tumor	Serve as novel biomarkers for diagnosing pancreatic cancer	(117)
tsRNA	tRF-Leu-CAG-002	Pancreatic tumor	Serve as novel biomarkers for diagnosing pancreatic cancer	(117)
tsRNA	tRNA-ValTAC-3	HCC	As a new biomarker for tumor diagnosis	(113)
tsRNA	tRNA-GlyTCC-5	HCC	As a new biomarker for tumor diagnosis	(113)
tsRNA	tRNA-ValAAC-5	HCC	As a new biomarker for tumor diagnosis	(113)
tsRNA	tRNA-GluCTC-5	HCC	As a new biomarker for tumor diagnosis	(113)
snRNA	U2snRNA	Acute leukemia	Lead to splicing errors	(67)

Although substantial progress has been achieved in the research of sncRNAs in tumor immunity, including notable advancements in using sncRNAs to improve the delivery of chemotherapeutic agents and counter tumor resistance and proliferation, cancer therapies based on sncRNAs still encounter several crucial challenges (29, 161). One of the foremost limitations of sncRNAs lies in their delivery systems, which significantly hinder clinical translation. Due to their negative charge and susceptibility to nuclease degradation, siRNAs face challenges in crossing the negatively charged cell membranes during systemic delivery. Furthermore, nonspecific uptake by non-target cells may trigger off-target effects or reduce therapeutic efficacy at the diseased site. Therefore, the development of efficient targeted delivery systems capable of transporting siRNA specifically to desired tissues is critical (161, 162). Although existing nanocarrier systems can address some of these issues, an ideal delivery platform should possess multiple essential features: protection of nucleic acid therapeutics, precise spatiotemporal control of drug release, selective targeting of specific cellular subpopulations within the TME, and efficient delivery under complex physiological conditions (62). For instance, in the context of osteosarcoma treatment, significant gaps remain in understanding the pharmacokinetics, metabolic pathways of nanocarriers, and long-term toxicity profiles (29). These knowledge gaps highlight the urgent need to establish a standardized evaluation framework for nanocarrier-based delivery systems (63).

Another major challenge is the limited understanding of the molecular mechanisms underpinning sncRNAs function, which restricts the refinement of therapeutic strategies. Although sncRNAs have been implicated in key processes such as immune checkpoint regulation and tumor-associated macrophage polarization, the dynamic regulatory networks involving sncRNAs within the TME have not yet been fully elucidated. In particular, the mechanisms of cross-talk between sncRNAs and epigenetic modifications, as well as those involving metabolic reprogramming, remain poorly understood. Additionally, synergistic or antagonistic interactions among different subclasses of sncRNAs require further investigation. Addressing these knowledge gaps will necessitate the application of advanced technologies such as single-cell sequencing and spatial transcriptomics.

More importantly, safety concerns remain a major barrier to the clinical application of sncRNA-based therapies. The toxicity mechanism of siRNA mainly includes its inherent immunogenicity, hybridization dependent toxicity (such as off target effect and targeted over silencing) and saturation effect of RNA interference mechanism. In addition, delivery systems (such as lipid nanoparticles and polymer carriers) may also trigger immune activation and chemical toxicity, such as complement activation, cell membrane damage and mitochondrial damage. To meet these challenges, researchers have adopted a variety of strategies: reducing the immunogenicity of siRNA by chemical modification (such as 2’-ome or 2’-f). Optimizing the seed zone design to reduce the Miss effect. Develop ionizable lipid nanoparticles to improve the safety of delivery systems. GalNAc coupling technology was used to achieve efficient liver targeting (163). The saturation of RNA interference mechanism was avoided by dose adjustment. In addition, the application of high-throughput toxicity screening and organ chip technology is helpful to evaluate the safety of siRNA and its vector more accurately. Although five kinds of siRNA drugs have been approved, their clinical transformation still needs to further solve the toxicity problem. In the future, it is necessary to combine artificial intelligence and biotechnology to optimize the design strategy to achieve safer and more efficient siRNA therapy (29, 163). Moreover, certain siRNA sequences may exhibit off-target effects. Notably, researchers have synthesized 2′-formamidonucleoside phosphoramidites as novel sugar-modified analogs for all four nucleobases, which effectively suppress off-target activity while enhancing *in vivo* stability (164).

As understanding of the roles of sncRNAs in tumor immunity deepens and nanotechnology-based delivery systems continue to evolve (33), the development of more efficient and safer sncRNA-based diagnostic and therapeutic strategies appears increasingly attainable. Artificial intelligence-driven platforms for nucleic acid drug design can enhance sequence specificity, while advances in organ-on-a-chip and digital twin technologies offer innovative tools for accurately evaluating therapeutic efficacy (165, 166). Given the critical roles of sncRNAs in the development and differentiation of immune cells, modulation of the TME, regulation of immune checkpoints, and facilitation of immune escape, as well as their potential as biomarkers, sncRNAs represent promising targets for integration with existing immunotherapies. When combined with approaches such as immune checkpoint inhibitors and CAR-T cell therapy, sncRNA-based interventions may offer novel strategies for early tumor diagnosis and immunotherapy, ultimately expanding treatment options and improving clinical outcomes for cancer patients.
